# Supplementary material for: Novel Assessment of Collagen and Its Crosslink Content in the Humerus from Primiparous Dairy Cows with Spontaneous Humeral Fractures Due to Osteoporosis from New Zealand
Source: Biology (Basel). 2022 Sep 23;11(10):1387. doi: 10.3390/biology11101387 (PMC9598166; doi:10.3390/biology11101387)
Supplement: Supplementary file 1 [file biology-11-01387-s001.zip › biology-1915472-supplementary.pdf]

biology MDPI

Table S1. Instrument configuration details and settings for collagen crosslink analysis.

LC system	Dionex UltiMate™ 3000 Rapid Separation Quaternary System (ThermoFisher Scientific, USA).
Mass spectrometer	Q Exactive™ Focus (ThermoFisher Scientific, Bremen, Germany)
Ionisation source	HESI-II (ThermoFisher Scientific, USA)
Analytical column	Cogent Diamond Hydride™ HPLC column, 2.2 μm particle size, 2.1 mm inner diameter, 150 mm length, 100 Å pore size (PM Separations NZ Ltd., Hamilton, New Zealand)
Flow rate	Analytical column: 0.4 mL/min
Column oven temperature	40 °C
Gradient	80–10% B for 3 min, 2.5 min hold at 10% B, 10–80% B for 1 min, 3 min equilibration at 80% B
Buffers	A: 0.1% Formic acid/water<br>B: 0.1% Formic acid/acetonitrile

Table S2. Mass spectrometer source settings for collagen crosslinks analysis and collagen crosslink detection.

**Crosslink Analysis**		**Crosslink Detection**	
Capillary temperature	350 °C	Resolution	35,000
S-Lens RF level	50%	Isolation window	1.0 *m*/*z*
Polarity	Positive	Default charge	1
Source voltage	4.0 kV	AGC target	2×10^4
Sheath gas flow rate	35 L/min	Maximum injection time	Auto
Aux gas flow rate	6 L/min	Minimum AGC target	8×10^3
Aux gas heater temperature	300 °C	Number of micro-scans	1
		Spectrum data type	Profile

Table S3. Inclusion list for parallel reaction monitoring analysis. The table shows accurate masses for all crosslinks DHLNL: dihydroxylysinonorleucine; HLNL: hydroxylysinonorleucine; HHL: histidinohydroxylysinonorleucine; HHMD: histidinohydroxymerodesmosine; DPD: deoxypyridinoline; PYD: pyridinoline.

**Crosslink**	**Mass (*m*/*z*)**	**CS [z]**	**Polarity**	**Start (min)**	**End (min)**	**CE**
DHLNL	308.1816	1	Positive	5.0	7.0	24
HLNL	292.1867	1	Positive	5.0	7.0	24
HHL	223.1237	1	Positive	3.0	7.5	20
HHMD	287.6635	2	Positive	5.0	8.5	12
DPD	413.2030	1	Positive	5.0	8.5	27
PYD	429.1979	1	Positive	5.0	8.0	30
